# Network Efficient Power Control for Wireless Communication Systems

**DOI:** 10.1155/2014/650653

**Published:** 2014-02-09

**Authors:** Daniel U. Campos-Delgado, Jose Martin Luna-Rivera, C. J. Martinez-Sánchez, Carlos A. Gutierrez, J. L. Tecpanecatl-Xihuitl

**Affiliations:** Facultad de Ciencias, Universidad Autónoma de San Luis Potosi, Avenue Salvador Nava s/n, Zona Universitaria, 78290 San Luis Potosi, SLP, Mexico

## Abstract

We introduce a two-loop power control that allows an efficient use of the overall power resources for commercial wireless networks based on cross-layer optimization. This approach maximizes the network's utility in the outer-loop as a function of the averaged signal to interference-plus-noise ratio (SINR) by considering adaptively the changes in the network characteristics. For this purpose, the concavity property of the utility function was verified with respect to the SINR, and an iterative search was proposed with guaranteed convergence. In addition, the outer-loop is in charge of selecting the detector that minimizes the overall power consumption (transmission and detection). Next the inner-loop implements a feedback power control in order to achieve the optimal SINR in the transmissions despite channel variations and roundtrip delays. In our proposal, the utility maximization process and detector selection and feedback power control are decoupled problems, and as a result, these strategies are implemented at two different time scales in the two-loop framework. Simulation results show that substantial utility gains may be achieved by improving the power management in the wireless network.

## 1. Introduction

As the demand for advanced mobile services increases, efficient use of network resources grows in importance. In a wireless network, services can have different requirements in terms of throughput or quality of service (QoS), which depend on the type of application: data, voice, or multimedia [1]. In this sense, the average bit error rate (BER) can be used as a measure of QoS given in terms of the signal to interference-plus-noise ratio (SINR) [2]. Typically, a mobile unit (MU) aims to attain a predefined SINR level in its transmission to the base station (BS) with the least power consumption. Moreover, it is desired to achieve this objective SINR level by allocating the network's resources in the most efficient way [3]. In cellular networks, the bandwidth or power efficiencies are the main criteria considered to measure the overall network performance [4–6]. Examples of solutions to this problem are given in [7–9], where the authors proposed game theoretic or optimization frameworks to maximize the users' utilities defined as a function of the transmission power.

From a cross-layer design principle for wireless networks [10], the resources allocation problem can be addressed more efficiently by sharing information among different layers [11, 12]. In this paper, we address the problem of improving the efficiency (profit) of a CDMA-based wireless network from the cross-layer perspective [13, 14]. A cross-layer approach is particularly important when designing protocols at the PHY and MAC link layers [15–17]. On the one hand, the PHY layer deals with data transmission over wireless channels gathering the units of modulation, SINR, channel coding, and so forth [15]; meanwhile, the data link layer has the objective of encoding bits into packets prior to transmission and decoding the packets back into bits at the destination.

In a CDMA-based network, cross-layer design has been addressed in the literature by the integration of units in the PHY layer such as multiuser detection, error correction, and channel estimation, with higher layers functions like power control, call admission control, packet collision resolution, and so on [18]. In [19], the cross-layer design problem of joint multiuser detection and power control is addressed. Similar to [7, 8], a game theoretic approach is proposed to maximize the users' utilities defined as the ratio of throughput and transmission power. All these approaches improve the users' utilities and consequently the network performance, by achieving a reduction in the power consumption or an increment in the throughput. However, to the best of the authors' knowledge, existing works on power control have been addressed following an energy-efficiency or spectral-efficiency criterion. The maximization of these performance metrics alone is not necessarily the best solution, since the effective cost of the resources required by the network might result in a commercial cellular network economically nonviable [20]. This problem arises because such definitions of the network's utilities focus on either energy efficiency or bandwidth efficiency regardless of the network cost.

The work by Akhtman and Hanzo [20] has shown that no significant economic gains can be obtained by just increasing the network's spectral efficiency (amount of bps/Hz). Furthermore, it is suggested that such economic gains may be possible when combining bandwidth augmentation and improving the power efficiency. In this paper, we explore this idea further by studying the open problem of how to maximize the network's utility (in monetary units) through the efficient use of the network resources. In particular, this paper seeks to maximize the network's utility (profit), as a function of the SINR metric, by considering adaptively the changes among three of the most valuable resources that are inherent in a mobile wireless network: (i) power consumption, (ii) effective bandwidth, and (iii) average user throughput. Furthermore, the proposed network efficient power control (NEPC) is based on a two-loop feedback configuration, as in [21, 22], where an outer-loop establishes the reference SINR by maximizing the network utility function, and also selects the optimal detector which minimizes the overall power consumption and an inner-loop achieves this reference SINR in all the MUs despite channel variations and roundtrip delays.

In this context, the major contribution of this work is to present a mechanism that manages to maximize the network's revenue and throughput by improving its power efficiency. This work focuses on a CDMA-based network [2, 23] such as direct-sequence CDMA (DS-CDMA) or multicarrier CDMA (MC-CDMA), since it comprises all varieties of systems impairments (multiple access interference, multipath, etc.) which makes a CDMA network more challenging than any other access techniques even those that are currently under consideration for 4G systems. However, and more importantly, the proposed mechanism can be extended to other access techniques, such as orthogonal frequency-division multiplexing (OFDM) [23, 24], as long as the SINR or SNR are employed as a QoS measure. Since any CDMA-based network capacity is interference limited [2, 23, 24], interference management is needed to maximize its performance. To manage the power consumption in the terminals, we formulate an overall power consumption index based on the whole power of the terminal and not just the radiated power, which makes this index strongly dependent on the detection strategy. Our implementation of the power consumption index is not found in the literature and is, therefore, a contribution of this work. Previous works [25, 26] have suggested preequalization to reduce interference and achieve a desired QoS. However, these schemes do not take into account the effects of processing/measurement delays and time-varying channels. Therefore, we consider feedback power control [27, 28] and detector selection as the main components in the proposed scheme not only to minimize the multiple-access interference (MAI), but also to improve the power efficiency in the network. Besides, this paper extends and complements our previous work in [29] with an analytical derivation of the utility optimization, a detailed presentation of the detector selection, and a completely new and comprehensive evaluation of the proposal.

The rest of the paper is organized as follows. In Section 2, the concept behind the proposed NEPC scheme is presented. The optimization strategies in the outer-loop are described in Section 3. First, the network utility concept is formulated from an economical viewpoint. Next, linear detectors such as matched filter (MF), zero-forcing (ZF), and minimum-mean-squared error (MMSE) are evaluated and compared with the purpose of selecting more efficiently the detector strategy in terms of both performance and power consumption.  Section 4 presents the feedback power control strategy implemented in the inner-loop, which is considered to achieve the required QoS in a distributed fashion. An extensive simulation evaluation is presented in Section 5 where the advantages of our proposal are highlighted. Finally, Section 6 presents the conclusions and future work.

The notation used in this paper is described next. *ℤ* denotes the set of integers, and ℝ and *ℂ* represent the real and complex numbers, respectively. ℝ^*N*^ and *ℂ* ^*N*^ stand for real and complex *N*-dimensional vectors. Scalars are represented by lower-case italic letters, and vector and matrices by boldface letters. (·)^*⊤*^ and (·)* describe the transpose and complex conjugate-transpose operators, respectively, and **I** denotes the identity matrix. For a vector **x** = [*x*_1_ ⋯ *x*_*N*_]^*⊤*^, the Euclidean norm is defined as $||x||=\sqrt{\sum_{i}{\left|x_{i}\right|}^{2}}$, and *ℰ*{·} is used to denote the expectation operator.

## 2. Proposed Network Efficient Power Control

**Figure 1:**
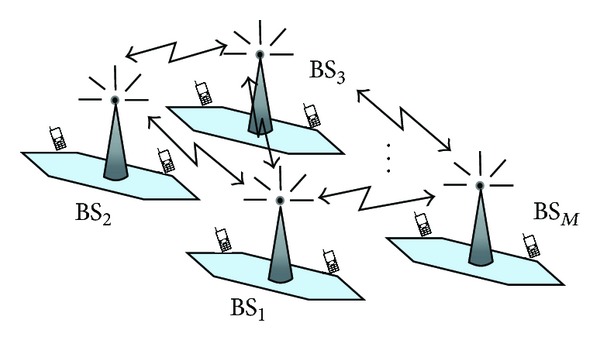
Structure of the wireless network.

In our formulation, the wireless network has *M* base stations (BS_1_,…, BS_*M*_), and each BS has *U*_*l*_ active MU's *l* = 1,…, *M* (see Figure 1). Hence, following network centric efficiency, we pursue to maximize the utility *𝒰* by optimizing the network power consumption *𝒫*, that is,

(1) $$\begin{array}{c} \underset{𝒫}{\max}𝒰. \end{array}$$

In this way, we assume that the network utility *𝒰* is affected by the power consumption in two major factors: (i) data transmission and (ii) hardware detection implementation [27, 30]. We assume that the power consumption of other stages at the BS, such as the RF section, is constant and, consequently, proportional to the power consumption of the detection method. Due to this reason, we just concentrate on the power demand by the detection stage. Nevertheless, this network centric optimization can be accomplished in a distributed fashion by recalling the results in [27, 31], where, for a given reference SINR, there exists only one solution to the minimum power allocation problem that achieves the reference in a given cell. In addition, we assume that the power consumption related to the signal processing and hardware employed by the detector in each BS is not affected by the neighbour BSs (no inter-cell interference), or this effect could be considered as thermal noise [2, 5]. Therefore, the NEPC scheme introduced here seeks to maximize the network utility while minimizing the power required in both transmission and detection implementation in each cell. Finally, the proposal focuses on power control at the uplink transmission, since we assume that the BSs transmit at a constant power in the downlink.

**Figure 2:**
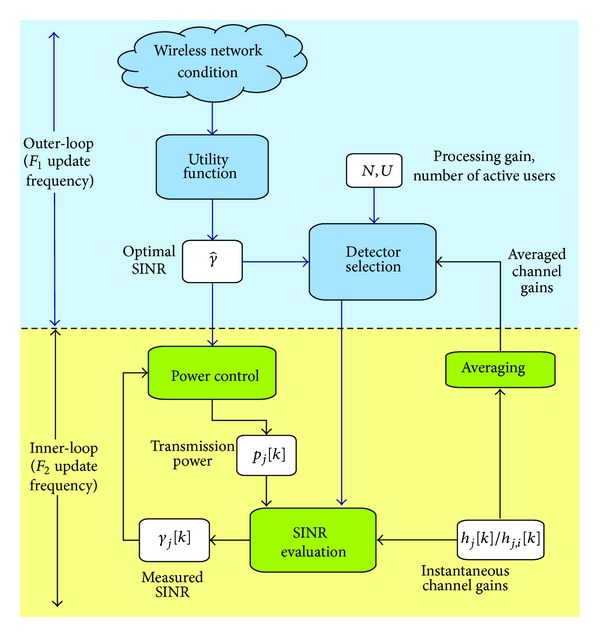
Two-loop interaction of the NEPC.

Overall, the proposed scheme considers three stages: (1) *network utility optimization*, (2) *detector selection*, and (3) *uplink closed-loop power control*. Note that, in practice, the network optimization process cannot be achieved in one step due to implementation limitations [32], such as information sharing, processing/measurement delays, and time-varying channel gains. Consequently, the proposed optimization scheme is carried out in a two-loop framework, as shown in Figure 2. In this schematic, there are two update frequencies involved: the first one for the utility function optimization and detector selection stages (*F*_1_) in the outer-loop, and the second update frequency for the feedback power control stage (*F*_2_) in the inner-loop, similarly to [21, 22]. For practical issues, the utility optimization process and detector selection run at a slower time scale than the feedback power control scheme; that is, *F*_1_ < *F*_2_. However, it is assumed that *F*_2_ is a multiple of *F*_1_ to facilitate the implementation; that is, *m*≜*F*_2_/*F*_1_ ∈ *ℤ*. On the other hand, the network optimization is focused on a CDMA-based network, where DS-CDMA and MC-CDMA are two important CDMA schemes for high rate wireless communications and therefore we aim our study on these schemes [2, 5, 23, 24]. Since the maximization problem will be solved in a distributed fashion, the index *l* related to each BS in the wireless network will be omitted. The general operation of the proposed NEPC scheme is as follows: the optimal SINR $\hat{\gamma }$ is calculated in the outer-loop to maximize the network utility function; after that, a linear detector is chosen by considering the number of active users in the cell *U*, optimal SINR $\hat{\gamma }$, averaged channel gains ${\overset{-}{h}}_{j}$ for DS-CDMA and ${\overset{-}{h}}_{j,i}$ for MC-CDMA, and processing gain *N*. The inner-loop is responsible for achieving $\hat{\gamma }$ at each BS by adjusting, in a feedback structure, the transmission power *p*_*j*_[*k*] of the MUs required to satisfy the restriction on the SINR of the *j*th user *γ*_*j*_ despite roundtrip delays, measurement noise, and channel variations. As is graphically described in Figure 2, the instantaneous values of the SINR are not employed by the outer-loop in our proposal; then, the roundtrip delays and measurement noise induced by processing or estimating the SINR at the BS do not affect the outer-loop structure. In addition, since this methodology involves sharing information between the PHY and MAC layers of the OSI model, our proposal establishes a cross-layer optimization [10]. In the next sections, we describe the structure of each stage in detail.

## 3. Outer-Loop Power Control

In this section, the network utility maximization and detector selection accomplished by the outer-loop in the NEPC are analytically derived.

### 3.1. Network Utility Optimization

The conventional utility concept refers to the level of satisfaction that each user experiences using a service [7, 21]. Since the air interface in a wireless communication represents a critical resource where the users have to consume battery energy to transmit information over it, these issues can be quantified conveniently by the following utility function:

(2) $$\begin{array}{c} u_{j}=\frac{T_{j}}{p_{j}},\;j=1,\ldots ,U, \end{array}$$

where *T*_*j*_ defines the throughput and *p*_*j*_ the transmission power per the *j*th user [8, 18]. Throughput is defined here as the number of information bits that are transmitted successfully in a given time period. Nonetheless, the utility described in (2) does not refer to monetary incentives; therefore, increasing (2) may not yield an economically viable network since the costs of energy and bandwidth are rapidly growing. Spectrum is a scarce and expensive resource and the power consumption cost constitutes a substantial factor in the financial bottom line of all major mobile carriers. In [20], a comprehensive analysis of a commercial wireless network is carried out by using the most basic economic principle, where Akhtman and Hanzo considered that the profit may be formulated as the revenue minus the actual cost of the provided services. Thus, the network utility per channel *𝒰*, in monetary units, can be formulated as [20]

(3) $$\begin{array}{c} 𝒰\left(\gamma \right)=\underset{\text{Revenue}}{\underset{︸}{\frac{1}{\overset{-}{B}}\log_{2}\left(1+\frac{\overset{-}{B}}{\overset{-}{R}}\log_{2}\left(1+\gamma \right)\right)}}-\underset{\text{Cost}}{\underset{︸}{\left(C_{p}5\phi N_{0}\gamma +\frac{C_{r}}{B}\right)}}, \end{array}$$

where $\overset{-}{B}=B/M$ and $\overset{-}{R}$ are the average bandwidth and the baseline data rate per user, *M* is the number of users in the global network, the coefficient *ϕ* denotes the transmission chain overall efficiency, *C*_*p*_ is the cost per Wattsecond by the carrier's network (Ws = joule), *C*_*r*_ is the cumulative rate of all additional costs not related to the power consumption, and *N*_0_ is the power spectral density (PSD) of the thermal noise. Finally, *B* denotes the network's effective total bandwidth, and *γ* is the averaged signal to interference-plus-noise ratio (SINR). In the original formulation by [20], *γ* is linked to the signal-to-noise ratio (SNR), but we propose to incorporate the effect of MAI in (3) by redefining *γ* as the SINR term, which is more appealing for multiaccess wireless systems. Nonetheless, depending on the studied wireless system, an approximated injective relation between SINR and SNR could be constructed by an estimation of the MAI in the system [2]. In terms of QoS evaluation, the SINR after signal detection is a better performance metric, since this parameter can be related to the frame-error rate (FER) or frame-success rate (FSR) of the transmission [33]. In fact, by using the set of parameters $\left\{M,\overset{-}{R},C_{p},\phi ,B,N_{0},C_{r}\right\}$ to define the wireless network condition, we can express the utility in (3) as a function of *γ*. Therefore, the following optimization problem is addressed:

(4) $$\begin{array}{c} \underset{\gamma }{\max}𝒰\left(\gamma \right). \end{array}$$

In the following, the partial derivatives of *𝒰* (for simplicity, we drop the dependency of *γ* in function *𝒰*) are derived in order to find the maximum with respect to *γ*:

(5) ∂𝒰∂γ=R−βB−2[1(1+γ)[1+βln⁡(1+γ)]−1α],∂2𝒰∂γ2=−R−B−2β(1+γ)2[1+βln⁡⁡(1+γ)]2×[1+β+βln⁡⁡(1+γ)],

where

(6) $$\begin{array}{c} \beta =\frac{\overset{-}{B}}{\ln\left(2\right)\overset{-}{R}},\;\;\;\alpha =\frac{1}{5\phi \overset{-}{R}C_{p}N_{o}{\left(\ln2\right)}^{2}}. \end{array}$$

Therefore, since $\left\{\overset{-}{B},\overset{-}{R},\alpha ,\beta \right\}$ are positive constants and the SINR is a positive value *γ* > 0, there exists a unique extreme point of *𝒰* that satisfies

(7) $$\begin{array}{c} {\frac{\partial 𝒰}{\partial \gamma }|}_{\gamma =\hat{\gamma }}=0\;⟹\;{\underset{f(\gamma )}{\underset{︸}{\alpha -(1+\gamma )}}|}_{\gamma =\hat{\gamma }}={\underset{g(\gamma )}{\underset{︸}{\beta (1+\gamma )\ln(1+\gamma )}}|}_{\gamma =\hat{\gamma }} \end{array}$$

since

(8) $$\begin{array}{c} f\left(0\right)>g\left(0\right),\;\;\frac{df}{d\gamma }<0,\;\frac{dg}{d\gamma }>0,\;\forall \gamma >0. \end{array}$$

As a result, the extreme point is a positive value $\hat{\gamma }>0$ such that $f(\hat{\gamma })=g(\hat{\gamma })$, and its uniqueness is guaranteed by the monotonic behaviour of *f*(·) and *g*(·) defined in (7) that imply a crossing point of both graphs. Furthermore, this extreme point is a maximum due to the concavity of *𝒰* for all *γ* > 0, since ∂^2^ *𝒰*/∂*γ* ^2^ < 0. However, an analytical solution to (4) is not viable, although its existence is guaranteed. So, we apply an iterative strategy based on Newton-Raphson method to find the SINR value that maximizes (3):

(9) $$\begin{array}{c} \gamma_{n+1}=\gamma_{n}-\left[\frac{\beta \left(1+\gamma_{n}\right)\ln\left(1+\gamma_{n}\right)-\alpha +\left(1+\gamma_{n}\right)}{\beta \ln\left(1+\gamma_{n}\right)+\beta +1}\right],\;n>0, \end{array}$$

which by the previous arguments will converge to $\gamma_{n}\to \hat{\gamma }$ independently of the initial condition *γ*_0_ > 0, as *n* → *∞*.

In this paper, we only consider the problem of maximizing an overall utility function of a multiuser CDMA-based wireless network where all users have a common SINR target; that is, the QoS is set the same for all users. The networks' utility maximization with multiple QoS requirements is beyond the scope of this paper and therefore is left for future work. Thus, once the common SINR target is estimated by the outer-loop, the inner-loop power control structure is responsible for regulating the transmission power for each active user in order to achieve the optimal SINR $\gamma_{j}=\hat{\gamma }$ for all *j* = 1,…, *U*. In addition, the selection of the detector provides a balance between the implementation complexity at the receiver and the transmission power given a QoS level. In fact, as will be shown in following sections, once the linear detector is fixed, there is only one power assignation rule that achieves the optimal SINR in the wireless system [27, 31], as long as, the resulting transmission power does not exceed the limitation of the transmission amplifier. Nonetheless, there is no coupling between the maximization process in (4) and the power control scheme, since both processes are independent, and this is a key property exploited by the NEPC structure in Figure 2.

### 3.2. Detector Selection

In the distributed optimization for network resources, each cell is studied independently which is based on a synchronous CDMA-based system with *U* active users. All users spread their data symbols at *k*-instant *b*_*j*_[*k*] ∈ *ℂ* for all *j* = 1,…, *U* with a specific sequence **c**_*j*_ = [*c*_*j*,1_ ⋯ *c*_*j*,*N*_]^*⊤*^ ∈ ℝ^*N*^ where $c_{j,i}=\{-1/\sqrt{N},1/\sqrt{N}\}$ for all *i* = 1,…, *N*. The load percentage of the CDMA-based system is defined as the ratio 100% × *U*/*N*. In this way, assuming users synchronization, the received signal **r**[*k*] at *k*-time instant in the BS (uplink transmission) after sampling and demodulation is given by [2, 5, 23]

(10) $$\begin{array}{c} r\left[k\right]=C\;\Gamma \left[k\right]b\left[k\right]+\eta \left[k\right], \end{array}$$

where, in the case of DS-CDMA, matrices **C** and Γ[*k*] are given by

(11) $$\begin{array}{c} C=\left[\begin{array}{ccc} c_{1} & \cdots & c_{U} \end{array}\right]=\left[\begin{array}{ccc} c_{1,1} & \cdots & c_{U,1}\\ \vdots & \ddots & \vdots \\ c_{1,N} & \cdots & c_{U,N} \end{array}\right]\in {\mathbb{R}}^{N\times U},\\ \Gamma \left[k\right]=\left[\begin{array}{ccc} h_{1}\left[k\right]\sqrt{p_{1}\left[k\right]} & & \\ & \ddots & \\ & & h_{U}\left[k\right]\sqrt{p_{U}\left[k\right]} \end{array}\right]\in {\mathbb{C}}^{U\times U}, \end{array}$$

where *h*_*j*_[*k*] ∈ *ℂ* and $\sqrt{p_{j}[k]}\in \mathbb{R}$ denote the channel gain and transmission power factor for the *j*th user, respectively; **b**[*k*] = [*b*_1_[*k*]  ⋯  *b*_*U*_[*k*]]^*⊤*^ ∈ *ℂ* ^*U*^ represents the vector of data symbols; each symbol *b*_*j*_[*k*] for all *j* = 1,…, *U* has zero mean and normalized energy, that is, *ℰ*{*b*_*j*_[*k*]} = 0 and *ℰ*{|*b*_*j*_[*k*]|^2^} = 1, and **η**[*k*] ∈ *ℂ* ^*N*^ represents a vector with zero-mean complex Gaussian noise components of variance *σ* ^2^ per dimension; that is, *ℰ*{**η**[*k*]} = 0, *ℰ*{**η**[*k*]*η[*k*]} = *σ* ^2^ **I**  ∀ *k*. For practical implications, all the power factors are restricted to a feasible interval; that is,

(12) $$\begin{array}{c} p_{\min}\leq p_{j}\left[k\right]\leq p_{\max},\;\forall j=1,\ldots ,U, \end{array}$$

where 0 < *p*_min⁡_ < *p*_max⁡_. Meanwhile, for a MC-CDMA system, the spreading codes matrix **C** is time varying, since the transmitters spread the data information over a set of subcarriers using the predefined spreading sequence **c**_*j*_ in the frequency domain (a chip of the spreading sequence is transmitted through each subcarrier) [24]; the matrices in (10) are described by

(13) $$\begin{array}{c} C=\left[\begin{array}{ccc} h_{1,1}\left[k\right]c_{1,1} & \cdots & h_{U,1}\left[k\right]c_{U,1}\\ \vdots & \ddots & \vdots \\ h_{1,N}\left[k\right]c_{1,N} & \cdots & h_{U,N}\left[k\right]c_{U,N} \end{array}\right]\in {\mathbb{C}}^{N\times U},\\ \Gamma \left[k\right]=\left[\begin{array}{ccc} \sqrt{p_{1}\left[k\right]} & & \\ & \ddots & \\ & & \sqrt{p_{U}\left[k\right]} \end{array}\right]\in {\mathbb{R}}^{U\times U}, \end{array}$$

where *h*_*j*,*i*_[*k*] ∈ *ℂ* denotes the channel gain for the *j*th user and *i*th subcarrier, *j* = 1,…, *U* and *i* = 1,…, *N*. To have an homogeneous notation for DS-CDMA and MC-CDMA, the explicit dependence on time in the spreading code matrix **C** is omitted for a MC-CDMA system.

For the data estimation process of the *j*th active user, a linear filter **χ**_*j*_ = [*χ*_*j*,1_ ⋯ *χ*_*j*,*N*_]^*⊤*^ ∈ ℝ^*N*^ is applied to the received signal [34]; that is,

(14) $$\begin{array}{c} {\hat{b}}_{j}\left[k\right]=\chi_{j}^{⊤}r\left[k\right],\;\forall j=1,\ldots ,U. \end{array}$$

Using **X** = [**χ**_1_ ⋯ **χ**_*U*_] ∈ ℝ^*N*×*U*^ to express the structure of the linear detector at the receiver [34], we address the following detection strategies [1, 27, 35]:

(i) matched filter (MF): **X** ^*⊤*^ = **C** ^*⊤*^;
(ii) zero forcing (ZF): **X** ^*⊤*^ = (**C** ^*⊤*^ **C**)^−1^ **C** ^*⊤*^;
(iii) minimum mean square error (MMSE):
  (15) $$\begin{array}{c} X^{⊤}={\left(C^{⊤}C+\sigma^{2}I\right)}^{-1}C^{⊤}. \end{array}$$

In this work, the linear detector **X** does not employ the transmission power information for data detection, in order to subsequently derive a tractable form of the SINR for closed-loop power allocation [27]. Consequently, the linear detector is constant and real in the case of DS-CDMA. Opposite to DS-CDMA, in MC-CDMA [23, 36], the linear detectors MF, ZF, and MMSE become complex and time-varying matrices, but their structure is analogous to DS-CDMA.

In general, a tradeoff between complexity and performance has to be considered for an efficient detector selection criterion. Next, we first analyse the complexity of the detectors which is defined in terms of the arithmetic operations at this level of design. These arithmetic operations are additions/subtractions and multiplications/divisions needed in the data estimation process. The complexity for additions is treated in the same way as subtractions. Also, the complexity of multiplications is considered the same as that of divisions.  Table 1 shows the evaluation of the complexity among the studied detectors: MF, ZF, and MMSE [37]. In order to estimate the complexity in the inverse of **C** ^*⊤*^ **C** and **C** ^*⊤*^ **C** + *σ* ^2^ **I** for the ZF and MMSE detectors, the Gauss-Jordan algorithm [38, 39] was applied. Although the MMSE detector requires the thermal noise variance estimation, this process is neglected for the complexity comparison.

Furthermore, power consumption is another efficiency metric of comparison at the implementation level [40]; then, the linear detectors' complexity must be expressed in terms of power consumption at circuit level. This is accomplished by calculating the total power dissipation of a CMOS design, *P*_total_ [41, 42]:

(16) $$\begin{array}{c} P_{\text{total}}=P_{\text{dynamic}}+P_{\text{static}}+P_{\text{leakage}}, \end{array}$$

where the static *P*_static_ and leakage *P*_leakage_ power dissipations are not considered at this level of design, since they are considered at the fabrication or synthesis process. Therefore, the dynamic power consumption *P*_dynamic_ is the only parameter considered, which is generated by the switching activity via the process of charging and discharging the load capacitance, which is defined by

(17) $$\begin{array}{c} P_{\text{total}}\approx P_{\text{dynamic}}=\theta CV_{\text{DD}}^{2}f, \end{array}$$

where *θ* represents a switching factor, *C* is the load capacitance, *V*_DD_ denotes the supply voltage, and *f* is the operation frequency. The switching factor *θ* depends on the used arithmetic logic, and this parameter can be considered as a constant in our evaluation. The supply voltage *V*_DD_ is also assumed fixed, and it could be viewed as a function of the semiconductor technology. The operation frequency is also fixed for comparison purposes. Therefore, the only parameter that is considered a function of the complexity is the load capacitance, and taking into consideration that the capacitance is calculated based on the number of transistors and their characteristics, we compute the complexity in terms of full adders, assuming that each one-bit full adder has eight transistors [43]. The number of full adders related to *X* additions is then expressed as *X* × *Y*, where *Y* is the number of bits used for calculations, and the equivalent number of full adders related to *Z* multiplications is (*Y* − 1)×(*Y* · *Z*); then, the circuit power consumption *P*_total_ as a function of number of full adders is given by

(18) $$\begin{array}{c} P_{\text{total}}=\theta \left[\vartheta \;Y\left(X+\left(Y-1\right)Z\right)WLC_{\text{gd}}\right]V_{\text{DD}}^{2}f, \end{array}$$

where *ϑ* is the number of transistors in the circuit, *W* defines the average transistor width and *L* its length, and *C*_gd_ denotes the gate plus diffusion capacitance; these three last parameters depend on the employed semiconductor technology. In this way, the complexity results in Table 1 can be used in conjunction with the power estimation in (18) to obtain an estimation of the required power in the implementation of the linear detectors in a CDMA-based system. For MC-CDMA, the only modification is related to parameter *Y*, since all the operations involve now complex numbers instead of real values.

### 3.3. Power Allocation

Power allocation is a common strategy to reduce the MAI component and extend battery life in cellular CDMA-based networks (uplink transmission). The objective of power allocation is to reach the required QoS or equivalently an objective SINR value *γ*_*i*_^obj^ for the *i*th user. Considering that the BS applies a linear detection strategy, the SINR can be expressed by [1, 27, 30]

(19) $$\begin{array}{c} \gamma_{j}=\frac{\delta_{jj}p_{j}}{\Sigma_{l\neq j}\delta_{jl}p_{l}+\omega_{j}\sigma^{2}},\;\forall j=1,\ldots ,U, \end{array}$$

where *p*_*j*_ is the power related to the *j* th user, *σ* ^2^ is the noise variance at the receiver, and (*δ*_*jj*_, *δ*_*jl*_, *ω*_*j*_) are constant positive parameters that depend on the applied linear detector (MF, ZF and MMSE) and spreading codes properties [30, 35, 36, 44]:

(20) $$\begin{array}{c} \delta_{j,l}=\{\begin{array}{cc} {\left|h_{j}\right|}^{2}{\left|\sum_{i=1}^{N}\chi_{j,i}c_{l,i}\right|}^{2} & \text{DS-CDMA}\\ {\left|\sum_{i=1}^{N}\chi_{j,i}c_{l,i}h_{l,i}\right|}^{2} & \text{MC-CDMA} \end{array}\\ \omega_{j}=\sigma^{2}\sum_{i=1}^{N}{\left|\chi_{j,i}\right|}^{2},\;\forall j,l=1,\ldots ,U. \end{array}$$

On the other hand, by assuming that the SINR per active user follows the value $\hat{\gamma }$ that maximizes the utility function *𝒰*, that is, $\gamma_{j}=\hat{\gamma }$ for all *j* = 1,…, *U*, the following vector equality is obtained from (19):

(21) $$\begin{array}{c} \frac{1}{\hat{\gamma }}\Gamma_{\text{BIT}}p=\Gamma_{\text{MAI}}p+\eta , \end{array}$$

where **p** = [*p*_1_ ⋯ *p*_*U*_]^*⊤*^ is the transmission power vector, matrices Γ_BIT_ and Γ_MAI_ contain the users' amplitudes and interference factors, and **η** denotes the noise vector. These matrices and vectors are defined as follows:

(22) $$\begin{array}{c} \Gamma_{\text{BIT}}≜\left[\begin{array}{ccc} \delta_{11} & & \\ & \ddots & \\ & & \delta_{UU} \end{array}\right]\in {\mathbb{R}}^{U\times U}, \end{array}$$

(23) $$\begin{array}{c} \Gamma_{\text{MAI}}≜\left[\begin{array}{cccc} 0 & \delta_{12} & \cdots & \delta_{1U}\\ \delta_{21} & 0 & \cdots & \delta_{2U}\\ \delta_{U1} & \delta_{U2} & \cdots & 0 \end{array}\right]\in {\mathbb{R}}^{U\times U}, \end{array}$$

(24) $$\begin{array}{c} \eta ≜\left[\begin{array}{c} \sigma^{2}\omega_{1}\\ \vdots \\ \sigma^{2}\omega_{U} \end{array}\right]\in {\mathbb{R}}^{U}. \end{array}$$

Consequently, the following centralized solution is obtained for the power allocation problem [27, 31]:

(25) $$\begin{array}{c} p_{o}={\left\{\frac{1}{\hat{\gamma }}\Gamma_{\text{BIT}}-\Gamma_{\text{MAI}}\right\}}^{-1}\eta \end{array}$$

provided that $\det((1/\hat{\gamma })\Gamma_{\text{BIT}}-\Gamma_{\text{MAI}})\neq 0$. In fact, this last condition is easily satisfied in practice for DS-CDMA systems, since in general the matrix $(1/\hat{\gamma })\Gamma_{\text{BIT}}-\Gamma_{\text{MAI}}$ is strictly diagonally dominant [45], since for orthogonal or low-correlation spreading codes *δ*_*jj*_ ≫ *δ*_*jl*_ for all *j*, *l* [27], and consequently, this matrix is nonsingular. Meanwhile, for MC-CDMA systems, this condition is not trivial, since the subcarrier channels destroy the orthogonality or low correlation among the spreading codes, so the nonsingularity of $(1/\hat{\gamma })\Gamma_{\text{BIT}}-\Gamma_{\text{MAI}}$ is difficult to fulfill for the MF detector, but in general feasible for the ZF and MMSE detectors [36].

The power solution in (25) is visualized as an open-loop solution, but it can only be used for reference purposes since the delays in the power allocation feedback paths and measurement uncertainty in the SINR quantification are not taken into account [46]. Nevertheless, (25) can provide a important reference for the transmission power employed by the MU's in the uplink transmission if it is calculated for the mean channel gains over a certain time window. Following this idea and considering an evaluation window of *m* samples at the update frequency *F*_2_, the averaged channel gains are given by

(26) $$\begin{array}{c} {\overset{-}{h}}_{j}=\frac{1}{m}\sum_{k=1}^{m}h_{j}\left[k\right]\;\text{DS-CDMA}, \end{array}$$

(27) $$\begin{array}{c} {\overset{-}{h}}_{j,i}=\frac{1}{m}\sum_{k=1}^{m}h_{j,i}\left[k\right]\;\text{MC-CDMA}, \end{array}$$

for *j* = 1,…, *U* and *i* = 1,…, *N*; next, these values are substituted in (22) to compute by (25) an estimation of the overall transmission power **p**_*o*_ in the examination period for the three linear detectors: MF, ZF, and MMSE.

### 3.4. Power Consumption Index

To manage the power consumption in the transmission of the MUs and detection process at the BS, we formulate the overall consumption index *J*_*q*_, which is given as a function of the detection circuit power *P*_total_^*q*^ and the transmission power **p**_*o*_^*q*^ given in (18) and (25), respectively, for each linear detector *q* = MF, ZF, and MMSE, as follows:

(28) $$\begin{array}{c} J_{q}=\underset{\text{Detection}}{\underset{︸}{\frac{P_{\text{total}}^{q}}{P_{\text{total}}^{q}+1}}}+\mu \underset{\text{Transmission}}{\underset{︸}{\frac{||p_{o}^{q}||}{||p_{o}^{q}||+1}},}\;q=\text{MF},\text{ZF},\text{MMSE}. \end{array}$$

The index *J*_*q*_ is calculated at a fixed *k* = *m*(*F*_2_/*F*_1_) instant (*m* ∈ *ℤ*) for the analysed linear detectors in the outer-loop; see Figure 2. In fact, the structure of (28) seeks to weight equally the power required by the implementation of the detector, and the transmission power of the MU's required to achieve the optimal SINR that maximizes the utility *𝒰*, since both the detection and transmission terms in *J*_*q*_ are positive values bounded by one. Hence, *μ* > 0 in the cost function (28) is selected to put more or less emphasis on the transmission power compared to the power consumption related to the complexity of the implementation. Therefore, the detector that minimizes (28) is selected until the next iteration of the outer-loop at *F*_1_ update frequency

(29) $$\begin{array}{c} \text{Linear}\;\text{Detector}=\arg\underset{q=\text{MF},\text{ZF},\text{MMSE}}{\min}J_{q}. \end{array}$$

Once the objective SINR, $\hat{\gamma }$, and the linear detector are selected, we employ in the inner-loop a feedback power control scheme to obtain the transmission power per user in a distributed fashion. Nonetheless, it is important to highlight that the information of the QoS quantification for each MU at the BS, which is expressed by the SINR measurements, is not employed by the outer-loop in our proposal (see Figure 2); then, the adverse effects of roundtrip delays and measurement noise do not affect the outer-loop structure.

## 4. Inner-Loop Power Control

**Figure 3:**
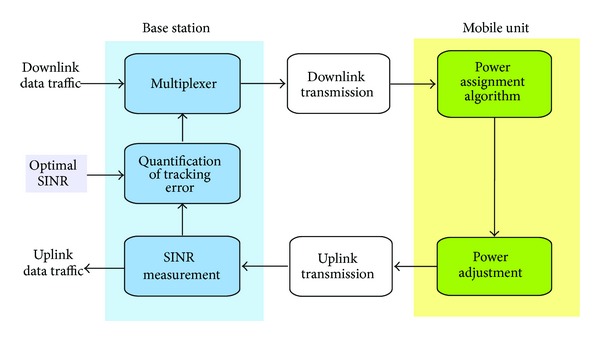
Inner-loop feedback power control for the uplink [47].

In this work, we adopt a control theory perspective to study the distributed power control [27, 47] in the inner-loop with the aim of analysing jointly the stability and reference tracking that can be achieved by using a feedback loop, as shown in Figure 3 for the uplink transmission. In this setup, the SINR estimation for each user *γ*_*j*_ is computed by the BS, and it is compared with the reference that achieves the desired QoS of the *j*th user *γ*_*j*_^obj^. In our proposal, the objective SINR is given by maximizing the utility *𝒰* in the outer-loop; that is, $\gamma_{j}^{\text{obj}}=\hat{\gamma }$ for all *j* = 1,…, *U*. Then, the base station transmits the following percentage error to the MU:

(30) $$\begin{array}{c} e_{j}\left[k\right]=\left(1-\frac{\gamma_{j}^{\text{obj}}}{\gamma_{j}\left[k\right]}\right)p_{j}\left[k\right],\;\forall j=1,\ldots ,U, \end{array}$$

where, based on this information, the power update is carried out. One important feature of this closed-loop power control is its robustness and capacity to handle time-varying channel gains [27]. A linear-quadratic (LQ) performance criterion is employed to derive the control law in [47]. Hence, the power update for the *j* th user at *k* th moment is formulated as

(31) p~j[k]=(1−Ω)pj[k−1]+Ωpj[k−nRT−1]−Ωaj[k−1],pj[k]=min⁡{pmax⁡,max⁡(p~j[k],pmin⁡)},

where now an orthogonal projection operator is proposed to the resulting transmission power after the LQ update to guarantee a solution inside the feasible interval in (12). In this way, the projected solution is used in the LQ recursive structure in order to reduce a windup effect by the integral action in the controller [48]. Furthermore, the received tracking error at the MU for the *j*th user is represented by

(32) $$\begin{array}{c} a_{j}\left[k\right]=e_{j}\left[k-n_{\text{RT}}\right], \end{array}$$

where *Ω* ∈ (0,1) is a control parameter and *n*_RT_ is the measurement and processing delay. Other distributed power control schemes, like Foschini-Miljanic [49], channel adaptive [50], variable-structure control [51], or switched proportional-integral-derivative control [52], could be also employed for this stage along with the orthogonal projector. However, the LQ control in (31) has the advantage of explicitly taking into the account the roundtrip delay *n*_RT_, besides its robustness described in [47].

In this way, the overall NEPC strategy described in the previous sections minimizes the power consumption as a function of the signal processing and hardware required by the linear detector and the transmission power of the mobile terminals and, at the same time, maximizes the utility of the wireless network by its two-loop structure and cross-layer interaction. Next, this strategy will be evaluated in detail for its three stages: network utility optimization, detector selection, and feedback power control.

## 5. Results

In this section, due to the similarities between the power control strategies in DS-CDMA and MC-CDMA, only a DS-CDMA network is used to evaluate the proposed two-loop NEPC introduced in Sections 2, 3, and 4. However, it is important to remark that the proposed cross-layer optimization scheme is general enough to be applied in other multiple-access techniques. First, the concavity of the utility function *𝒰* in (3) is verified numerically, along with the existence of a maximum in practical conditions. Next, the dependence of the power consumption index *J*_*q*_ in (28) on the load of the system, objective SINR, and linear detectors is analysed through a Monte Carlo evaluation. Finally, the NEPC structure in Figure 2 is implemented for two load conditions and variable objective SINR to illustrate the importance of the detector selection, and hence a cross-layer interaction in the CDMA-based system.

### 5.1. Utility Evaluation

We evaluate the DS-CDMA system performance by using the statistics of a commercial wireless network reported in [20].  Table 2 shows the characteristics of the cellular network which are used for the simulations. The channel gain at the *k* th iteration is modeled with the following time-varying profile [53]:

(33) $$\begin{array}{c} {\left|h_{j}[k]\right|}^{2}=g_{j}\left[k\right]Y_{j}^{-1}\left[k\right],\;\forall j=1,\ldots ,U, \end{array}$$

where log⁡(*Y*_*j*_[*k*]) is a random Gaussian variable with zero mean and variance 0.1 and *g*_*j*_[*k*] is also a random variable but with a Rayleigh distribution [5].

**Figure 4:**
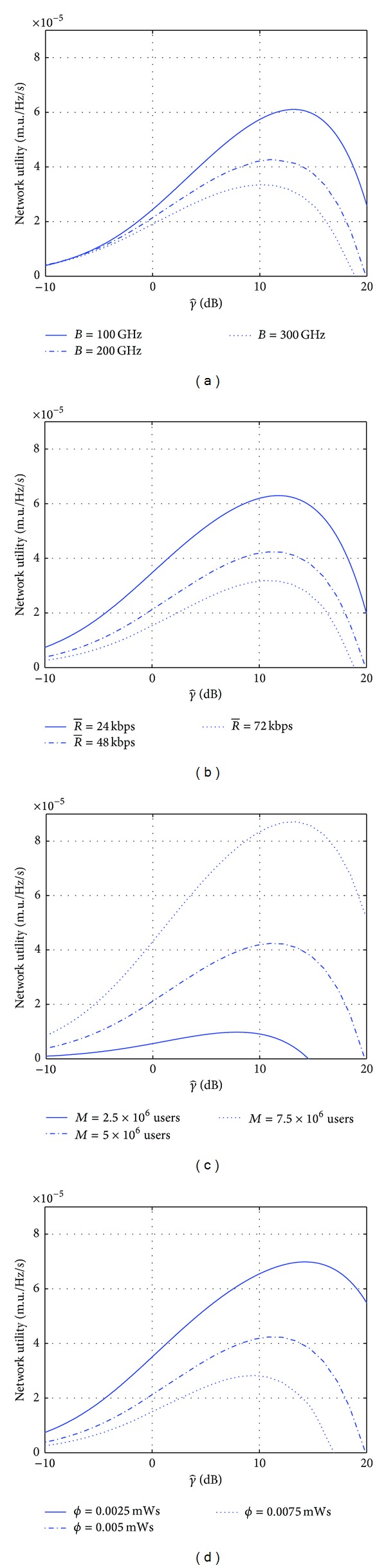
Network utility versus average SINR for the parameters in Table 2 and applying ±50% variation in the nominal values of ($B,\overset{-}{R},M,\phi$).

**Figure 5:**
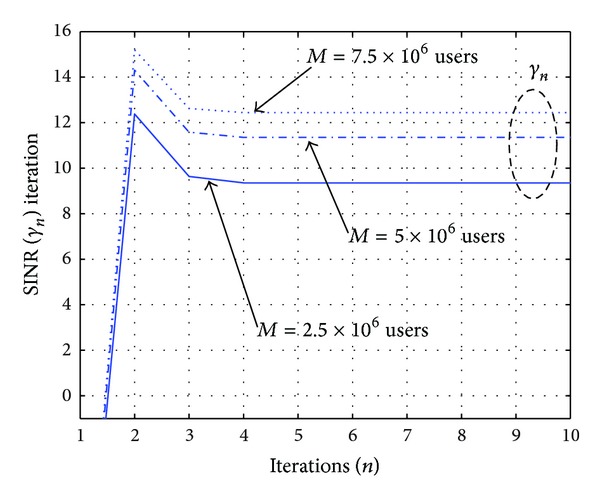
Newton-Raphson sequence for the maximum SINR of the network utility by considering *M* = 2.5 × 10^6^, 5 × 10^6^, and 7.5 × 10^6^ users.

Figure 4 displays the utility function defined in (3) as a function of the averaged SINR given the information in Table 2, and also applying a ±50% variation on 4 parameters $\left\{B,\overset{-}{R},M,\phi \right\}$. As a consequence, in all the plots, a clear maximum is visualized for the network utility with respect to the SINR *γ*, as expected by the analysis presented in Section 3.1. When applying the iterative Newton-Raphson method to find the SINR value that maximizes (3), the convergence is reached in less than 5 iterations. In order to illustrate this property, the SINR iteration *γ*_*n*_ obtained by applying (9) with an initial condition *γ*_0_ = 0.01 is illustrated in Figure 5 for three conditions *M* = 2.5 × 10^6^, 5 × 10^6^ and 7.5 × 10^6^ users. The convergence points of these sequences in Figure 5 are described in Table 3. Without losing generality, these three conditions will be considered as testing cases in the next evaluation for the detector selection and feedback power control in the inner-loop.

### 5.2. Detector Selection

As illustrated in Figure 2, once the optimal SINR $\hat{\gamma }$ is calculated, the next step in our proposal is to perform the linear detector selection in the outer-loop and apply the feedback power control scheme in the inner-loop, with the aim of attaining the optimal SINR with the minimum power consumption. The power consumption index in (28) is evaluated by assuming a CDMA system with a processing gain of *N* = 32, normalized *m*-sequence spreading codes, and varying the load of the system from 1% to 100%. The weight *μ* in (28) is set to 100 to indicate more emphasis in the transmission power than in circuit implementation. However, a further analysis is required to estimate a precise value for the parameter *μ*, since the implementation costs for the transmission components and the processing circuit are not the same. Nonetheless, this parameter is tuned according to actual hardware considerations in the transmission/detection process. A Monte Carlo evaluation is carried out with 5,000 realizations of the channel gains in (33). The first step in this evaluation is to measure the complexity as a function of load capacitance by using the characteristics of a 65 nm technology transistor, as shown in Table 4 [54]. We define a 16-bit full adder to obtain the circuit power consumption based on (17) with a switching factor of *θ* = 0.2, and frequency of *f* = 140 MHz. The operation frequency at the receiver processing is assumed twice [55]. Notice that this way of calculating the circuit power represents just an initial approach; further reduction in power consumption is possible by considering the architecture design of the circuit. Subsequently, the total transmission power **p**_*o*_ for the three detectors (MF, ZF, and MMSE) is obtained through (25) for all the channel realizations.

**Figure 6:**
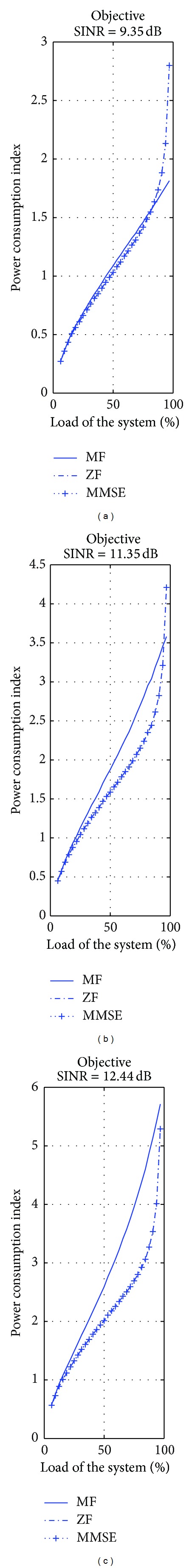
Power consumption index for MF, ZF, and MMSE detectors as a function of the load of the CDMA system: (a) $\hat{\gamma }=9.35\;$dB, (b) $\hat{\gamma }=11.35\;$dB, and (c) $\hat{\gamma }=12.44\;$dB.

Figure 6 shows the resulting power indices for three optimal SINRs $\hat{\gamma }$: 9.35, 11.35, and 12.44 dB (see Table 3). In this way, Figure 6 shows important average tendencies: (a) the ZF and MMSE both have a similar performance for any load condition, (b) the ZF and MMSE have a lower power consumption index for loads lower than 85–90%, and (c) the index behaviour is dependent on the objective SINR and load conditions. As a consequence, these ideas strengthen our proposal of selecting in an adaptive fashion the linear detector, as will be shown next.

### 5.3. Inner-Loop Evaluation

**Figure 7:**
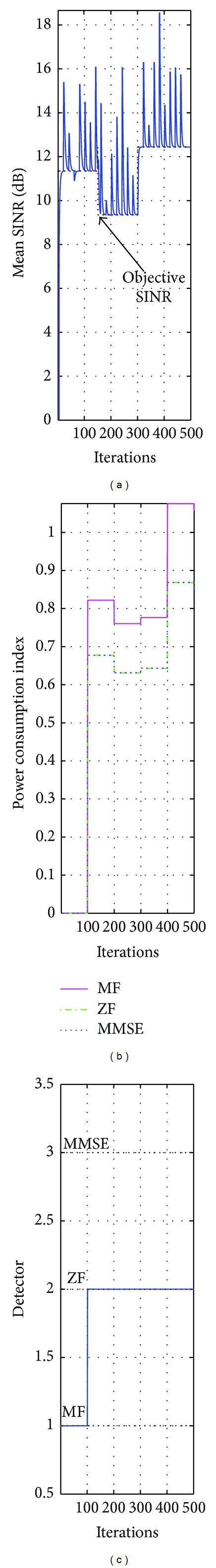
Closed-loop response of NEPC algorithm for a 50% load: (a) averaged SINR among active users dB, (b) power consumption index, and (c) selected linear detector.

**Figure 8:**
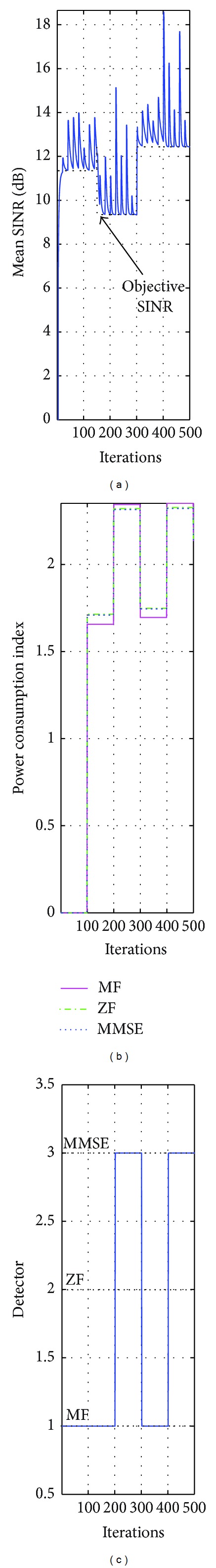
Closed-loop response of NEPC algorithm for a 100% load: (a) averaged SINR among active users dB, (b) power consumption index, and (c) selected linear detector.

The final step in our proposal is to apply the feedback power control scheme in the inner-loop described in Section 4. A distributed algorithm is employed by applying the LQ controller in (31) whose parameters are given in Table 5. In order to show the adaptive structure of the NEPC scheme, we consider that the network conditions result in the following optimal SINR's:

- $k<150\Rightarrow \hat{\gamma }=11.35\;$dB,
- $150\leq k<300\Rightarrow \hat{\gamma }=9.35\;$dB, and
- $k\geq 300\Rightarrow \hat{\gamma }=12.44\;$dB,

where *k* denotes the time index. In this simulation, the evaluation window is 500 iterations, where in each 100 steps the detector performance is evaluated (*m* = 100 in (26)) by the criterion in (29). In this way, by considering *F*_1_ = 1, 500 Hz as the frequency of power control update [56], then optimization and detector selection are carried out at a frequency *F*_2_ = 15 Hz. Figures 7 and 8 show the resulting performance by considering 50% and 100% loads. At the beginning of the simulation, the MF is the default detector until the first evaluation at iteration 100. Both figures show that the LQ power allocation scheme can adjust the transmission power to achieve the optimal SINR despite the channel variations. In Figure 7, the power consumption index selects the ZF after the first evaluation step; meanwhile, Figure 8 illustrates that the MF and MMSE can be both selected in this scenario. These results are in agreement with the average tendencies in Figure 6. Consequently, Figures 7 and 8 show the importance of considering the adaptive NEPC scheme in Figure 2, since the network resources could be chosen in an efficient way. As mentioned earlier, the same strategy could be applied for MC-CDMA wireless systems; however, as pointed out in [36], just the ZF and MMSE are available choices for the detector, since the MF cannot overcome the interference caused by the equivalent time-varying spreading codes [23].

## 6. Conclusions

In this paper, we proposed a NEPC scheme that maximizes the profit of a wireless network based on a two-loop framework and cross-layer optimization. By recalling that, for a reference SINR of the MU in the cell, there exists a unique transmission power that achieves this objective [31], the network utility maximization is carried out in a distributed fashion. Hence the NEPC scheme relies on the periodic maximization of the utility function and detector selection according to the networks conditions in the outer-loop, allowing achieving the QoS with the minimum resources in the transmission/detection process in the uplink. Hence, the proposed strategy combines two optimization stages in the outer-loop and a feedback power control in the inner-loop to maximize the network utility. A key property in the proposal is that the utility maximization process and feedback power control scheme are decoupled problems, and, as a result, both strategies can be implemented at two different time scales (see Figure 2). Results show that the NEPC proposal not only guarantees the maximum profit of the network but also improves its power efficiency. As future work, it is suggested to study the effect of more unpredictable channels than those reported in this work. In addition, it is our interest to formulate the problem of a network that supports various services.

## Figures and Tables

**Table 1:** Complexity evaluation of different linear detectors.

Detector	Additions/subtractions (*X*)	Multiplications/divisions (*Z*)
MF	(*N* − 1) *U*	*NU*
ZF	*U* ^3^ + *NU* ^2^ + *NU* − 2*U*	2 (*U* ^3^ + *U* ^2^ + *NU*) + *NU* ^2^
MMSE	*U* ^3^ + *U* ^2^ (*N* + 1) + *NU* − 2*U*	2 (*U* ^3^ + *U* ^2^ + *NU*) + *NU* ^2^

**Table 2:** Wireless network characteristics [20].

Parameter	Value
*B*	200 MHz
$\overset{-}{R}$	48 kbps
*C*_*p*_	0.0028 p/kWs
*N*_0_	10 dBm
*ν*	2.5
*M*	5 × 10^6^ users
*ϕ*	0.005 mWs
*C*_*r*_	50% of revenue
*N*_*f*_	7

**Table 3:** Optimal values for the utility maximization.

Optimum SINR	*M*		
	2.5 × 10^6^ users	5 × 10^6^ users	7.5 × 10^6^ users
$\hat{\gamma }$	9.35 dB	11.35 dB	12.44 dB

**Table 4:** Characteristics of A 65 NM technology transistor.

Parameter	Value
*V*_DD_	1.0 V
*C*_gd_	1.8*fF*/*μ*m
*W*	12*λ*
*L*	0.025 *μ*m/*λ*

**Table 5:** Simulation parameters for the inner-loop feedback power control.

Parameter	Value
*N*	32
*p*_min⁡_	1 pW
Ω	0.4
*σ* ^2^	−10 dBm
*p*_max⁡_	500 mW
*n*_RT_	2

## References

[1] Martınez-Lopez FJ, Campos-Delgado DU, Luna-Rivera JM, Arce-Santana E Quality-of-service analysis for linear multiuser detectors in the uplink of a wireless network.

[2] Proakis JG (2001). *Digital Communications*.

[3] Da B, Ko CC (2012). Dynamic resource allocation in relay-assisted OFDMA cellular system. *European Transactions on Telecommunications*.

[4] Wang C, Chan KY (2013). Utility-based admission control for mobile WiMAX networks. *Wireless Networks*.

[5] Rappaport TS (2002). *Wireless Communication Principles & Practice*.

[6] Goot R R, Trigano T, Tapuchi S, Gavan J (2013). Adaptive allocation of power transmission for high-altitude platforms. *Annals of Telecommunications*.

[7] Saraydar CU, Mandayam NB, Goodman DJ (2002). Efficient power control via pricing in wireless data networks. *IEEE Transactions on Communications*.

[8] Meshkati F, Chiang M, Poor HV, Schwartz SC (2006). A game-theoretic approach to energy-efficient power control in multicarrier CDMA Systems. *IEEE Journal on Selected Areas in Communications*.

[9] Elmusrati M, Jäntti R, Koivo HN (2007). Multiobjective distributed power control algorithm for CDMA wireless communication systems. *IEEE Transactions on Vehicular Technology*.

[10] Perez-Neira AI, Campalans MR (2008). *Cross-Layer Resource Allocation in Wireless Communications: Techniques and Models from PHY and MAC Layer Interaction*.

[11] Hu C (2011). Efficient cross-layer protocol for bandwidth-satisfied multicast in multi-rate MANETs. *Wireless Networks*.

[12] Lu J, Ma M (2011). Cross-layer MAC protocol and holistic opportunistic scheduling with adaptive power control for QoS in WiMAX. *Wireless Personal Communications*.

[13] Jiang H, Zhuang W, Shen X (2005). Cross-layer design for resource allocation in 3G wireless networks and beyond. *IEEE Communications Magazine*.

[14] You L, Wu P, Song M, Song JD, Zhang Y (2011). Cross-layer optimisation for uplink transmission in OFDMA cellular networks with fixed relays. *European Transactions on Telecommunications*.

[15] Miao G, Himayat N, Li Y, Swami A (2009). Cross-layer optimization for energy-efficient wireless communications: a survey. *Wireless Communications and Mobile Computing*.

[16] Popovski P, Ingram MA, Peel CB, Hara S, Toumpis S (2009). Cross-layer design for the physical, MAC, and link layer in wireless systems. *Eurasip Journal on Advances in Signal Processing*.

[17] Zhang Y, Leung C (2009). Cross-layer resource allocation for real-time services in OFDM-based cognitive radio systems. *Telecommunication Systems*.

[18] Buzzi S, Poor HV, Saturnino D (2009). Adaptive cross-layer distributed energy-efficient resource allocation algorithms for wireless data networks. *Eurasip Journal on Advances in Signal Processing*.

[19] Meshkati F, Poor HV, Schwartz SC, Mandayam NB (2005). An energy-efficient approach to power control and receiver design in wireless data networks. *IEEE Transactions on Communications*.

[20] Akhtman Y, Hanzo L (2010). Power versus bandwidth efficiency in wireless communications: from economic sustainability to green radio. *China Communications*.

[21] Chen BS, Yang CY, Li SY (2008). Adaptive two-loop power tracking control in CDMA systems with the utility optimization. *IEEE Transactions on Wireless Communications*.

[22] Yang CY, Chen BS, Jian CY (2012). Robust two-loop power control for CDMA systems with multiobjective optimization. *IEEE Transactions on Vehicular Technology*.

[23] Fazel K, Kaiser S (2008). *Multi-Carrier and Spread Spectrum Systems: From OFDM and MC-CDMA to LTE and WiMAX*.

[24] McCormick AC, Al-Susa EA (2002). Multicarrier CDMA for future generation mobile communication. *Electronics and Communication Engineering Journal*.

[25] Bisaglia P, Sanguinetti L, Morelli M, Benvenuto N, Pupolin S (2005). Pre-equalization techniques for downlink and uplink TDD MC-CDMA systems. *Wireless Personal Communications*.

[26] Cosovic I, Schnell M, Springer A (2005). Combined equalization for uplink MC-CDMA in Rayleigh fading channels. *IEEE Transactions on Communications*.

[27] Campos-Delgado DU, Luna-Rivera M (2012). Unified framework for the analysis and design of linear uplink power control in CDMA systems. *Wireless Networks*.

[28] Luna-Rivera JM, Campos-Delgado DU (2013). Distributed power control with multiuser detection for asynchronous DS-CDMA networks subject to time-delays. *Telecommunication Systems*.

[29] Martinez-Sanchez C, Luna-Rivera JM, Campos-Delgado DU A cross-layer power allocation scheme for CDMA wireless networks.

[30] Catrein D, Mathar R (2008). Feasibility and power control for linear multiuser receivers in CDMA networks. *IEEE Transactions on Wireless Communications*.

[31] Mathar R, Schmeink A (2008). Proportional QoS adjustment for achieving feasible power allocation in CDMA systems. *IEEE Transactions on Communications*.

[32] Gunnarsson F, Gustafsson F (2003). Control theory aspects of power control in UMTS. *Control Engineering Practice*.

[33] Dey S, Evans J Optimal power control in wireless data networks with outage-based utility guarantees.

[34] Verdu S (1998). *Multiuser Detection*.

[35] Campos-Delgado DU, Martínez-López FJ, Luna-Rivera JM (2007). Analysis and performance evaluation of linear multiuser detectors in DS-CDMA systems applying spectral decomposition. *Circuits, Systems and Signal Processing*.

[36] Campos-Delgado DU, Luna-Rivera JM (2013). Optimal pre-equalization for wireless MC-CDMA systems under QoS requirements. *IET Communications*.

[37] Comaniciu C, Mandayam NB, Poor HV (2005). *Wireless Networks: Multiuser Detection in Cross-Layer Design*.

[38] Grossman SI (1994). *Elementary Linear Algebra*.

[39] Trefethen LN, Bau D (1997). *Numerical Linear Algebra*.

[40] Li T, Ma C, Li W (2013). The system power control unit based on the on-chip wireless communication system. *The Scientific World Journal*.

[41] Weste NHE, Eshraghian K (1994). *Principles of CMOS VLSI Design—A System Perspective*.

[42] Marković D, Stojanović V, Nikolić B, Horowitz MA, Brodersen RW (2004). Methods for true energy-performance optimization. *IEEE Journal of Solid-State Circuits*.

[43] Chowdhury SR, Banerjee A, Roy A, Saha H (2008). A high speed 8 transistor full adder design using novel 3 transistor XOR gates. *International Journal of Electrical and Computer Engineering*.

[44] Campos-Delgado DU, Luna-Rivera JM (2013). Closed-loop pre-equalization for wireless MC-CDMA Systems under QoS requirements. *Journal of Franklin Institute*.

[45] Horn RA, Johnson CR (1985). *Matrix Analysis*.

[46] Gunnarsson F, Gustafsson F, Blom J (2001). Dynamical effects of time delays and time delay compensation in power controlled DS-CDMA. *IEEE Journal on Selected Areas in Communications*.

[47] Campos-Delgado DU, Luna-Rivera JM, Martínez-López FJ (2010). Distributed power control algorithms in the uplink of wireless CDMA systems. *IET Control Theory and Applications*.

[48] Goodwin GC, Graebe SE, Salgado ME (2001). *Control System Design*.

[49] Foschini GJ, Miljanic Z (1993). Simple distributed autonomous power control algorithm and its convergence. *IEEE Transactions on Vehicular Technology*.

[50] Choi SO, You KH (2008). Channel adaptive power control in the uplink of CDMA systems. *Wireless Personal Communications*.

[51] Uykan Z, Koivo HN (2006). Variable structure power control algorithm in mobile radio systems. *IEEE Transactions on Wireless Communications*.

[52] Safonov MG, Paul A, Akar M, Mitra U (2005). Adaptive power control for wireless networks using multiple controllers and switching. *IEEE Transactions on Neural Networks*.

[53] Fan X, Alpcan T, Arcak M, Wen TJ, Başar T (2006). A passivity approach to game-theoretic CDMA power control. *Automatica*.

[54] Feng Z (2010). *EE4800 CMOS Digital IC Design & Analysis*.

[55] Cheung TC (1998). *2GHz W-CDMA radio transceiver [M.S. thesis]*.

[56] Lee S (2000). *Spread Spectrum CDMA: IS-95 and IS-2000 for RF Communications*.

